# Precipitation phenomena in Al-Zn-Mg alloy matrix composites reinforced with B_4_C particles

**DOI:** 10.1038/s41598-017-10291-4

**Published:** 2017-08-29

**Authors:** Chuandong Wu, Kaka Ma, Dalong Zhang, Jialu Wu, Shuya Xiong, Guoqiang Luo, Jian Zhang, Fei Chen, Qiang Shen, Lianmeng Zhang, Enrique J. Lavernia

**Affiliations:** 1 State Key Laboratory of Advanced Technology for Materials Synthesis and Processing, Wuhan University of Technology, Wuhan, 430070 PR China; 2 Department of Chemical Engineering and Materials Science, University of California-Irvine, Irvine, CA 92697 USA; 3 Department of Mechanical Engineering, Colorado State University, Fort Collins, CO 80523 USA; 4 Oak Ridge National Laboratory, 1 Bethel Valley Rd, Oak Ridge, TN 37831 USA

## Abstract

To provide insight into precipitation phenomena in age-hardening Al-Zn-Mg(-Cu) matrix composites, an Al 7075 alloy composite reinforced with B_4_C particles was selected as a model system. The bulk composites were fabricated via plasma activated sintering and followed by a peak aged (T6) heat treatment. Two types of Al matrix zones were identified in the composite: (1) the regions in the vicinity of the matrix/reinforcement interface, defined as “matrix plastic zone” (MPZ) hereafter, and (2) the regions away from the matrix/reinforcement interface, simply defined as matrix hereafter. The precipitation behavior in the MPZ was characterized and compared to that in the matrix. The MPZ contained a high density of dislocations. The number density of GP zones in the MPZ is lower than that in the matrix while the average size of the GP zones in MPZ is coarser. In addition, semi-coherent platelet η′ precipitates were observed but only in the MPZ. The dislocations and the Al/B_4_C interfaces provide more heterogeneous nucleation sites for the η′ precipitates in the MPZ. The growth and coarsening of the η′ precipitates caused rapid depletion of Mg and Zn solute atoms in the MPZ.

## Introduction

Al-based metal matrix composites (MMCs) containing ceramic particle reinforcements are of interest partly due to the fact that they can be processed using flexible approaches, including: powder metallurgy, preformed infiltration as well as a variety of casting technologies^1–3^. Review of the published literature shows that interest in precipitation hardened, Al-based MMCs stems from their technological potential, as well as from the underlying scientific questions associated with these materials^4–7^. On one hand, an age-hardened Al alloy matrix has the potential to further increase the strength of the composite beyond the strengthening contribution from the reinforcement phase; on the other hand, the aging kinetics in the matrix alloy are influenced by the presence of the reinforcement particles which leads to interesting questions^5, 6, 8–11^. For example, how are aging kinetics influenced by the characteristics of the reinforcement particles (i.e., chemistry, size and distribution), the processing method, and the heat treatment conditions^12, 13^?

Amongst age-hardening Al alloys, Al-Zn-Mg(-Cu) alloys have been the subject of extensive research in the past decades due to their inherent high strength and stiffness^8^. The precipitation behavior in this alloy family is rather complicated, especially during the early stages, because of its sensitivity to the local chemical composition of the alloys and the heat treatment environment. Nevertheless, it is generally accepted that the precipitation sequence is the same for most of the Al-Zn-Mg alloys, and can be summarized as follows^14–18^:

Supersaturated solid solution (SSS) → Guinier-Preston zone (GP zone, coherent Mg and Zn-rich clusters) → η′ (semi-coherent MgZn_1−2_) → equilibrium η (incoherent MgZn_2_, hexagonal structure).

Some studies hypothesized that the precipitation sequence in the unreinforced matrix and that in the corresponding composites are identical^5, 19^. As a result, in an effort to promote precipitation, composites with age-hardening Al alloy matrix are usually given identical heat treatments as those used for the corresponding age-hardening Al alloys, including solution treatment, quenching, and aging. Interestingly, one critical factor that affects precipitation behavior is the role of dislocations, which has been neglected in most studies. Despite the fact that most of the dislocations are annihilated when the materials are heated during solution heat treatment, dislocations reappear and emanate from the matrix/reinforcement interface during quenching/cooling due to the mismatch in the coefficient of thermal expansion (CTE) between the reinforcement and the matrix^20–22^. These thermally-induced dislocations lead to the formation of a punching-out zone or matrix plastic zone (MPZ) around each reinforcements^5, 23, 24^. In contrast, matrix regions that are located away from these interfaces are considered to be unaffected by the CTE mismatch^25^. The relationship between dislocations and precipitation phenomena is well established. For example, Legros *et al*. observed the “pipe diffusion” phenomenon of Si solute in Al matrix by *in-situ* TEM. The experimental results indicated that dislocations can accelerate precipitate growth because atomic transport is kinetically faster in the presence of dislocations relative to bulk diffusion in a crystal^26^. Ma *et al*. also reported that the coupling of dislocations and precipitates in Al-Zn-Mg(-Cu) alloy by *in-situ* TEM, and concluded that dislocation motion could drag solute atoms to heterogeneous nucleation sites of η′ precipitates and accelerate the growth and coarsening of the precipitates at the dislocation cores^15^. Moreover, Hu *et al*. reported that the amount of heterogeneously nucleated precipitates varied within an Al 7093/B_4_C composites fabricated by the Boralyn technique^8^. However, there is a lack of fundamental information on the mechanisms that govern the precipitation behavior in Al-Zn-Mg (-Cu) alloy matrix composites. Precipitation is unique to this family of age-hardenable Al alloys and significantly contributes to their mechanical properties. As the research interest in the Al composites based on age-hardenable Al alloy matrix grows^27–29^, it is vital to develop an in-depth understanding of the precipitation mechanisms when reinforcing phase is added to age-hardenable Al alloy to form composites.

In view of the above, the objective of the present study is to provide insight into the following questions: First, is precipitation in the MPZ fundamentally different from that in the matrix? If there is a difference, how do dislocations influence the type, size, morphology and distribution of precipitates? Second, how does the presence of dislocations affect the nucleation and growth of precipitates in the composites? To provide insight into these questions, Al 7075 alloy reinforced with B_4_C particles was selected as a model system. Accordingly, bulk composites were consolidated by plasma activated sintering (PAS). This consolidation method was selected because it involves relatively low temperatures, which are required to avoid the formation of undesirable phase transformations and reactions in B_4_C reinforced Al composites^30–32^.

## Results

**Figure 1:**
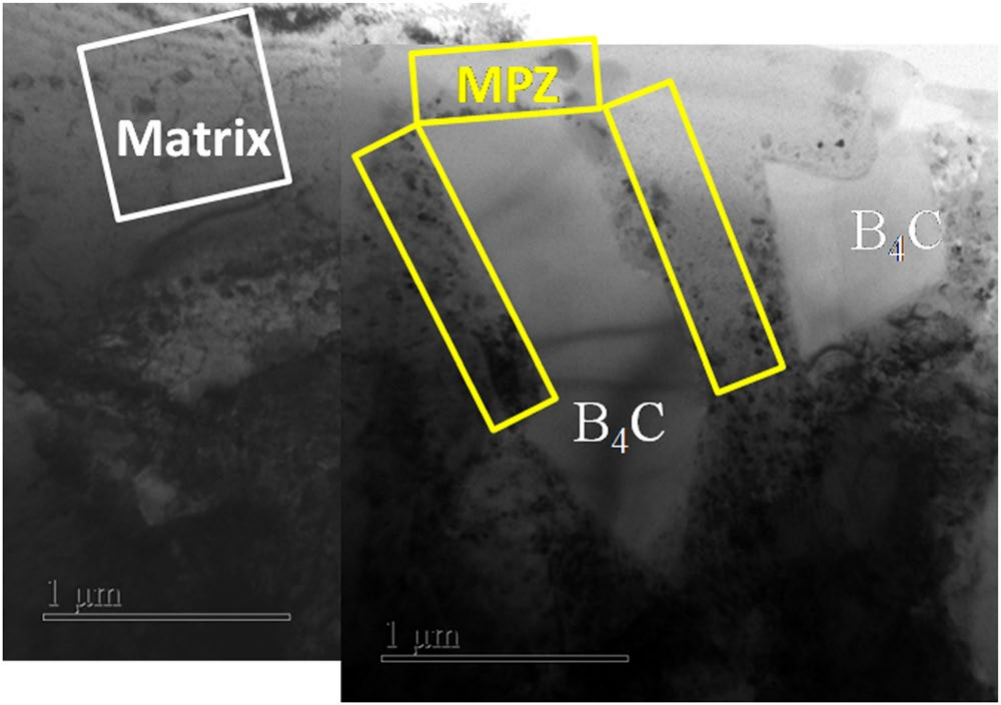
Bright-field TEM image of Al 7075/B_4_C composites showing the matrix and matrix plastic zone (MPZ), the B_4_C particles have an angular morphology containing straight and sharp interface with the matrix alloy. A variety of precipitates are observed in the matrix (marked by white box) and MPZ (marked by yellow box).

The size, distribution and chemistry of precipitated phases in the Al 7075/B_4_C composite were studied using TEM, and the results are shown in Fig. 1. As evident in Fig. 1, the B_4_C particles have an angular morphology characterized by straight and well defined interfaces with the matrix; this is consistent with the characteristics of the as-received B_4_C particles as reported in our previous research^33^. Details on the distribution and characteristics of the various precipitates in different regions (the matrix and MPZ) are described in this section.

### Precipitation in the matrix

**Figure 2:**
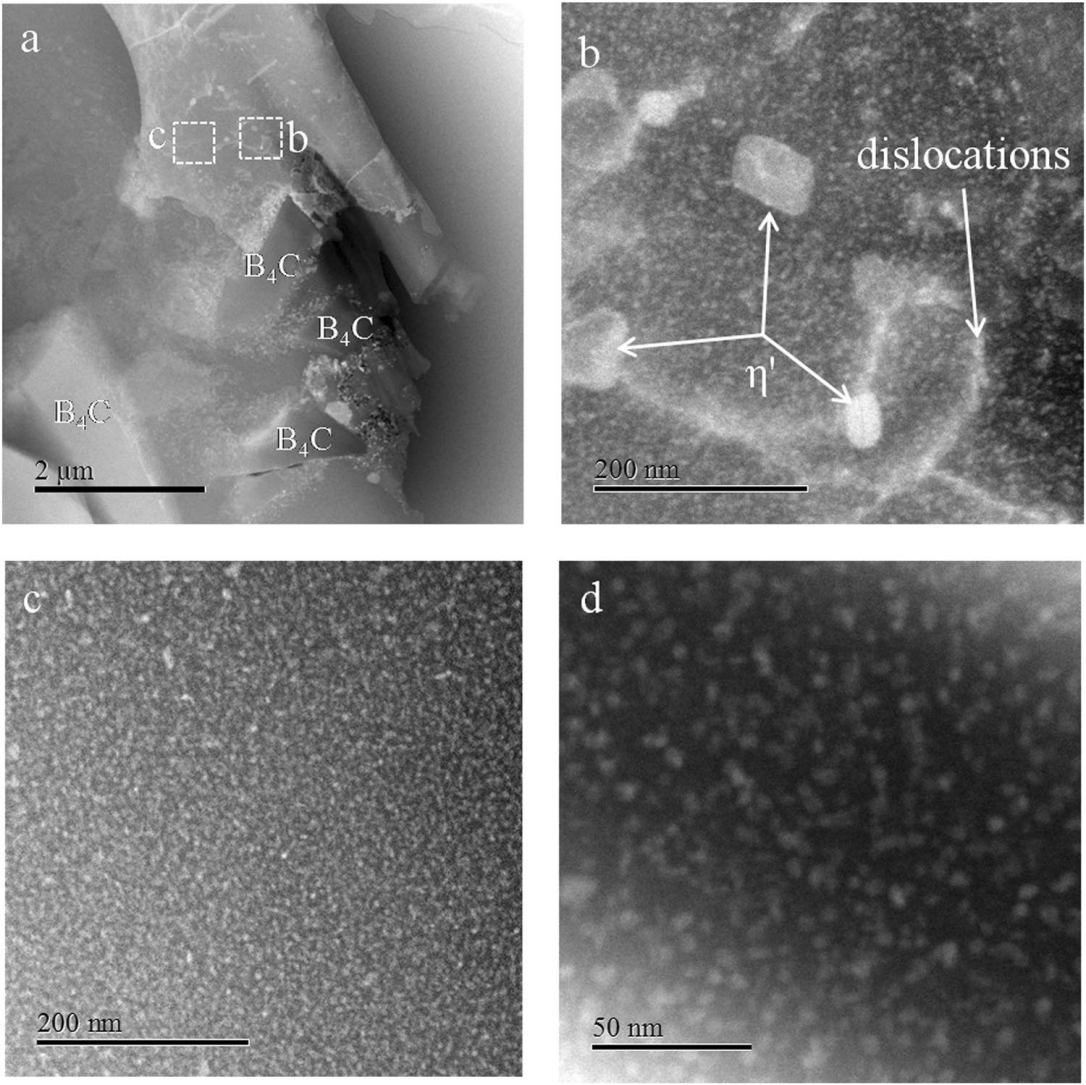
STEM images of precipitates in the matrix showing the distribution and morphology of the precipitates. (**a**) GP zones and plate-like η′ precipitates are observed as the predominant precipitates, which are commonly observed in T6 tempered Al-Zn-Mg alloys. (**b**) An enlarged image of one portion in (**a**), the majority of the plate-like η′ precipitates (diameter ~58.2 nm) are distributed on or near the dislocation lines the dislocation lines in a typical grain interior. (**c**,**d**) The high number density of GP zones (diameter ~3.7 nm) distribute homogeneously in the matrix.

**Figure 3:**
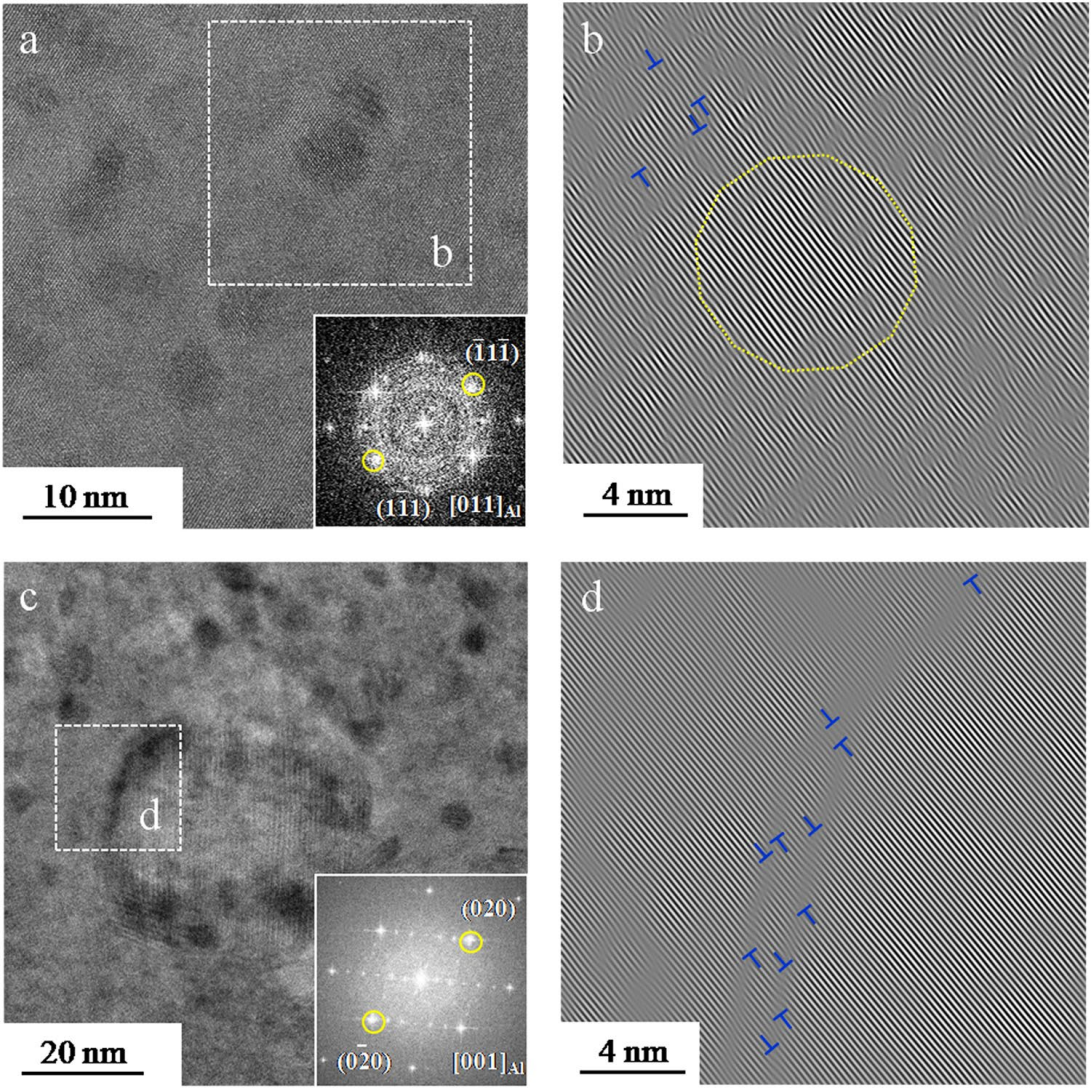
(**a**) HRTEM of spherical GP zone in the matrix, (**b**) Corresponding inverse FFT pattern of outlined area in (**a**,**c**) HRTEM of plate-like η′ in the matrix, the inset image shows the corresponding FFT pattern of the outlined area; (**d**) corresponding inverse FFT pattern of outlined area in (**c**).

The scanning transmission electron microscope (STEM) images in Fig. 2 show the microstructure of the composite and the precipitates that are present in the Al matrix. Heat treatment did not completely anneal all the dislocations, as shown in Fig. 2b, and dislocation lines are observed in a typical grain interior. The predominant precipitates are identified as GP zones and plate-like η′ precipitates according to their morphology, size, and structural coherency with the Al matrix reported in refs 15, 16 and 34 and the electron diffraction patterns are shown in Fig. 3. The majority of the plate-like η′ precipitates (diameter ~58.2 nm) are distributed on or near dislocation lines. This can be related to the fact that the dislocations act as the favorable nucleation sites for the η′ precipitates^34^.

In addition to the plate-like η′ precipitates in the matrix, a high number density of GP zones (diameter ~3.7 nm) are also found in Fig. 2b–d (white spherical precipitates). The size and number density of GP zones around dislocations exhibit no difference relative to those within the Al matrix, indicating that dislocations do not act as preferential sites for heterogeneous nucleation of GP zones in the matrix. Moreover, the presence of the fine spherical GP zones distributed randomly in the matrix alloy after quenching suggests that they are predominantly formed by homogeneous nucleation from a highly supersaturated solid solution^14, 35^.

The first precipitate phase in the precipitation sequence that can be detected via TEM is GP zones^36, 37^. It is evident in Fig. 3a that GP zones appear as dark spots regardless of grain orientation in a bright field image. GP zones are structurally coherent with the Al matrix with a very small lattice mismatch. The corresponding inverse Fast Fourier transform (FFT) images (Fig. 3b) in outlined area of Fig. 3a clearly confirm the presence of coherent lattice planes. Careful observation of the lattice fringes revealed the absence of dislocations in the GP zones, while edge dislocations are observed with several nanometers away from GP zones (highlighted by the symbol “⊥”). In addition to GP zones, the plate-like phase is identified as η′ precipitates from high resolution TEM micrographs, as illustrated in Fig. 3c. The corresponding inverse FFT image in Fig. 3d shows the distribution of the dislocations at the interface between the matrix and the plate-like precipitates, suggesting that the plate-like η′ precipitates is semi-coherent with the face center cubic Al lattice.

### Precipitation in the MPZ

**Figure 4:**
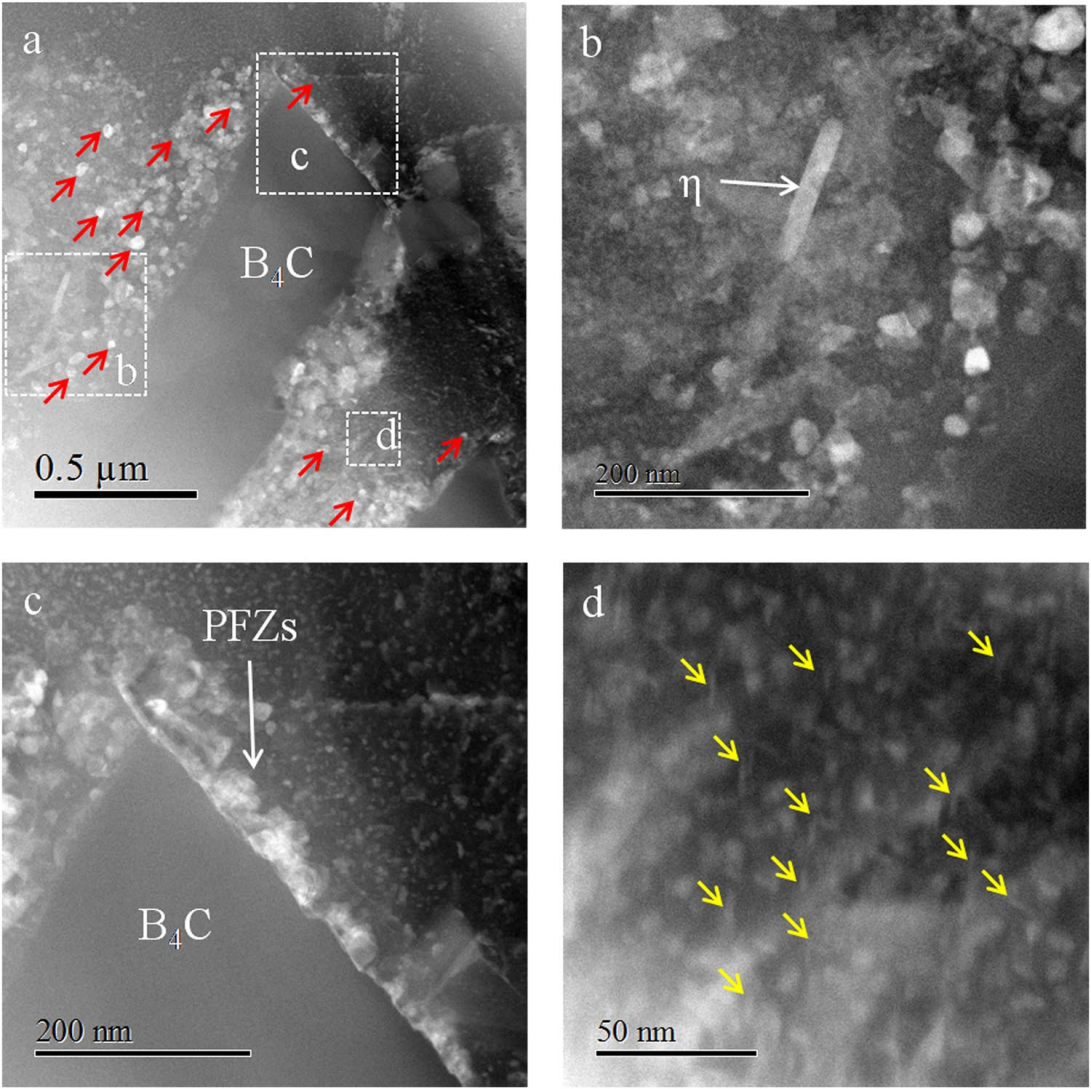
Representative STEM images of precipitates in the MPZ showing the distribution of various precipitates: lath-like η (**b**), plate-like η′ precipitates (**a**,**b**), GP zones (**c**,**d**) and platelet η′, precipitates free zones (PFZ) are also found around the B_4_C particles (**c**). The plate-like η′ precipitates are marked by red arrows in (**a**), while the platelet η′ precipitates are marked via yellow arrows in (**d**).

Figure 4 contains a series of STEM images illustrating the microstructure and precipitates in the MPZ. Compared to the matrix, one critical difference in the MPZ is that various precipitates are detected in the vicinity of B_4_C particle via STEM. Figure 4 shows a high number density of plate-like η′ precipitates (diameter ~25.1 nm) in the vicinity of B_4_C particles. The small white spherical clusters are GP zones (diameter ~4.7 nm), while the fine platelet precipitates are typically η′ precipitates (11.4 nm in length and 2.1 nm in width). In addition to these predominant phases commonly observed in T6 tempered Al 7075 alloy, several lath-like η precipitates were also found near the Al 7075/B_4_C interface with an average size of 110.4 nm in length and 22.0 nm in width (Fig. 4b). In addition, precipitate-free zones can be observed near the Al/B_4_C interface as shown in Fig. 4c. Both GP zones and platelet η′ precipitates are uniformly distributed in the MPZ. The number density of GP zone in the matrix and the MPZ are 7.98 × 10^9^ mm^−2^ and 5.58 × 10^9^ mm^−2^, respectively. Comparing Figs 4d and 2d, it is evident that the number density of GP zones in MPZ is lower than that in matrix, while the size of the precipitates in MPZ is slightly coarser.

**Figure 5:**
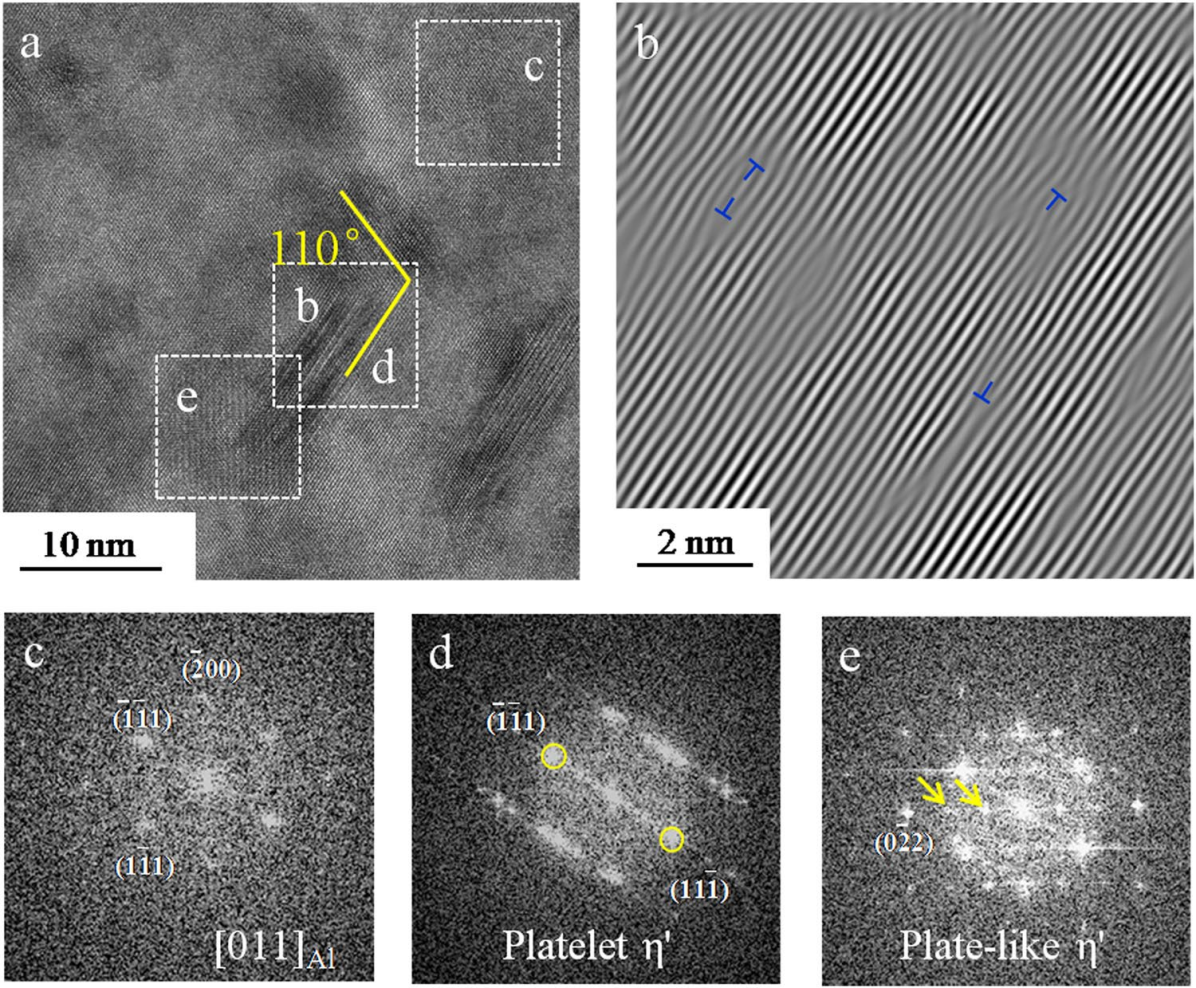
(**a**) HRTEM of the platelet η′ and the plate-like η′ in the MPZ, (**b**) corresponding inverse FFT pattern of outline area in (**a**) showing the dislocation in the vicinity of the platelet η′, (**c**–**e**) corresponding FFT patterns of outlined area in (**a**) showing the matrix, platelet η′ and the plate-like η’, respectively. The spots of the plate-like η′ were marked via yellow arrows in (**e**).

Figure 5 shows GP zones, platelet η′ and plate-like η′ that were identified via high resolution TEM as well as their morphology, size, and structural coherency with the Al matrix according to published studies^15, 16, 34, 38^. The spherical GP zones with dark contrast are observed in the area. It is generally accepted that GP zones are less stable than η′ precipitates, and that GP zones transform to platelet η′ precipitates due to the high concentration of solute atoms^14^. The detection of the platelet η′ precipitates in Fig. 5a support this point. Some prior studies have confirmed that the platelet η′ precipitates formed via the transformation from GP zones^34, 39^. The primary habit planes of platelet η′ in Al-Zn-Mg alloy are {111} planes. The platelet η′ precipitates grow in both (‐1‐11) and (‐11‐1) plane in [011]_Al_ zone axis. The angle between the two platelet η′ precipitates observed in Fig. 5a is approximately 110°, which is close to that of the angles (109.5° or 70.5°) between the (‐1‐11) and (‐11‐1) plane. Moreover, Fig. 5d shows the presence of streaking parallel to (11‐1)_Al_, which is associated with the η′ transition phase. As shown in Fig. 5e, the plate-like η′ precipitates spots at 1/3 or 2/3 of {220} were also clearly observed in [011]_Al_ zone axis. Interestingly, the plate-like η′ is observed to be in contact with the platelet η′ precipitate. Thus, it is probable that platelet η′ precipitates, which can be considered as an intermediate phase, eventually coarsen and transform into the metastable η′^14, 40^.

**Figure 6:**
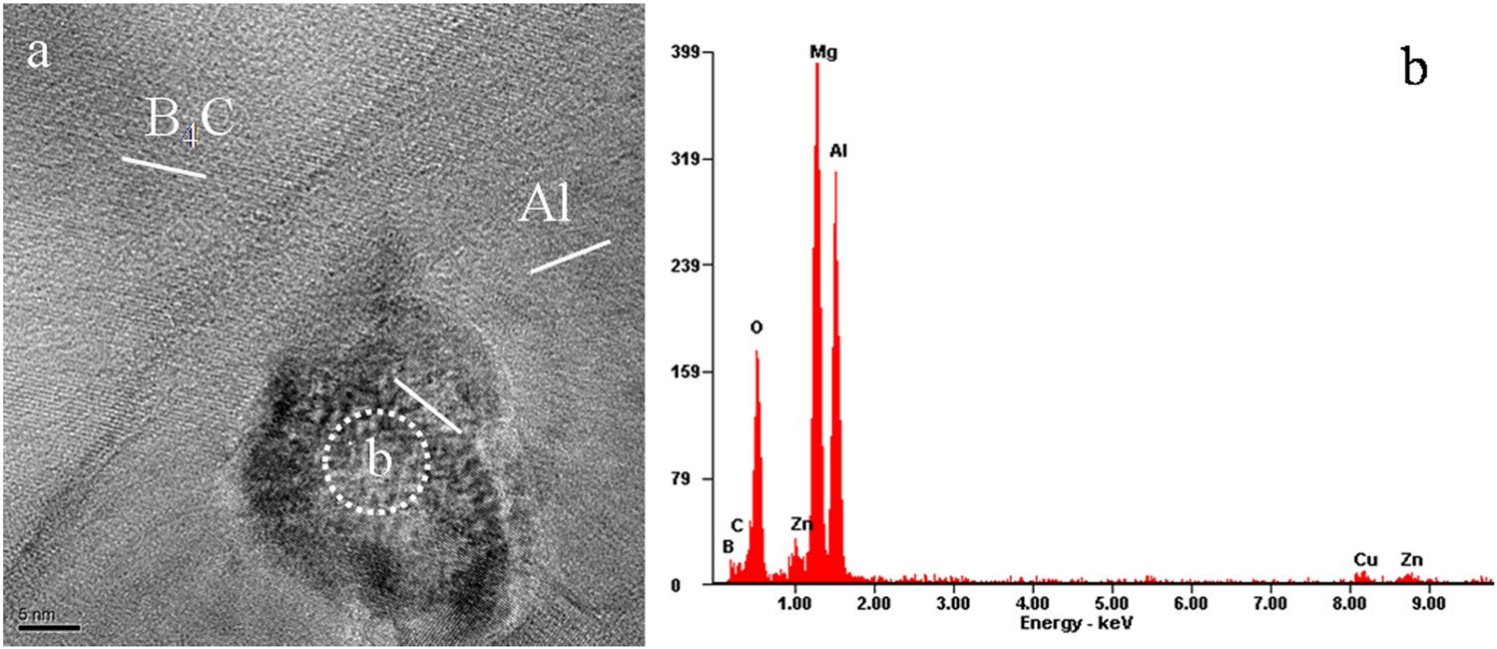
(**a**) HRTEM of the interface of Al 7075/B_4_C and (**b**) EDS providing the qualitative measurement of composition of the dispersed phase.

In addition, one incoherent particle was found by HRTEM near the Al alloy/B_4_C interface in Fig. 6. An EDS spectrum was taken on the particle, as shown in Fig. 6b, indicating that the particle contains a high concentration of Mg, Al and O. Thus, it is probable that these particles are MgO or MgAl_2_O_4_, which are commonly observed in Al alloys. Several previous studies^8, 41^ reported that Mg segregation phenomenon at Al alloy/B_4_C interfaces could lead to the formation of MgO or MgAl_2_O_4_ layer. Details regarding Mg segregation behavior, including the distribution and formation mechanism, can be found elsewhere^42^. Mg segregation at Al/B_4_C interfaces may also cause the depletion of the matrix solute atoms.

**Figure 7:**
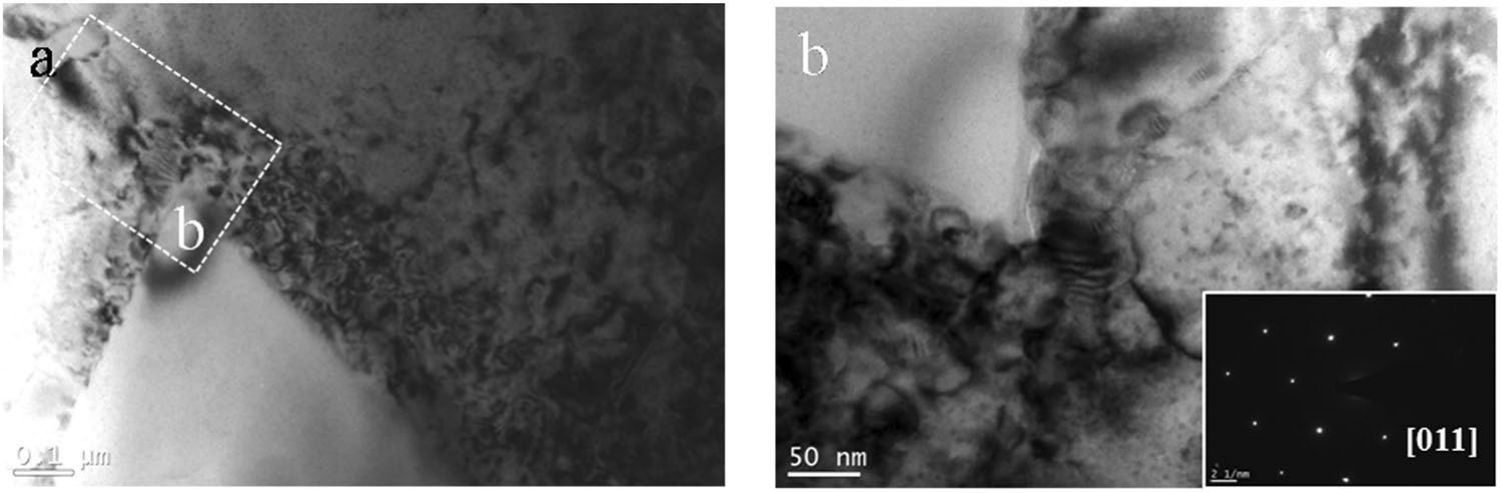
Bright-field image in the vicinity of the B_4_C particles showing the dislocation density in the MPZ is higher relative to that in the matrix, (**b**) An enlarged image of one portion of (**a**) showing the distribution of dislocations in the vicinity of B_4_C particle.

The distribution of dislocations in the vicinity of the B_4_C particles was characterized in the [011]_Al_ zone axis, as shown in Fig. 7. It is clear that the dislocation density in the MPZ is higher than that in the matrix, as evidenced by the enlarged image in Fig. 7b.

## Discussion

**Figure 8:**
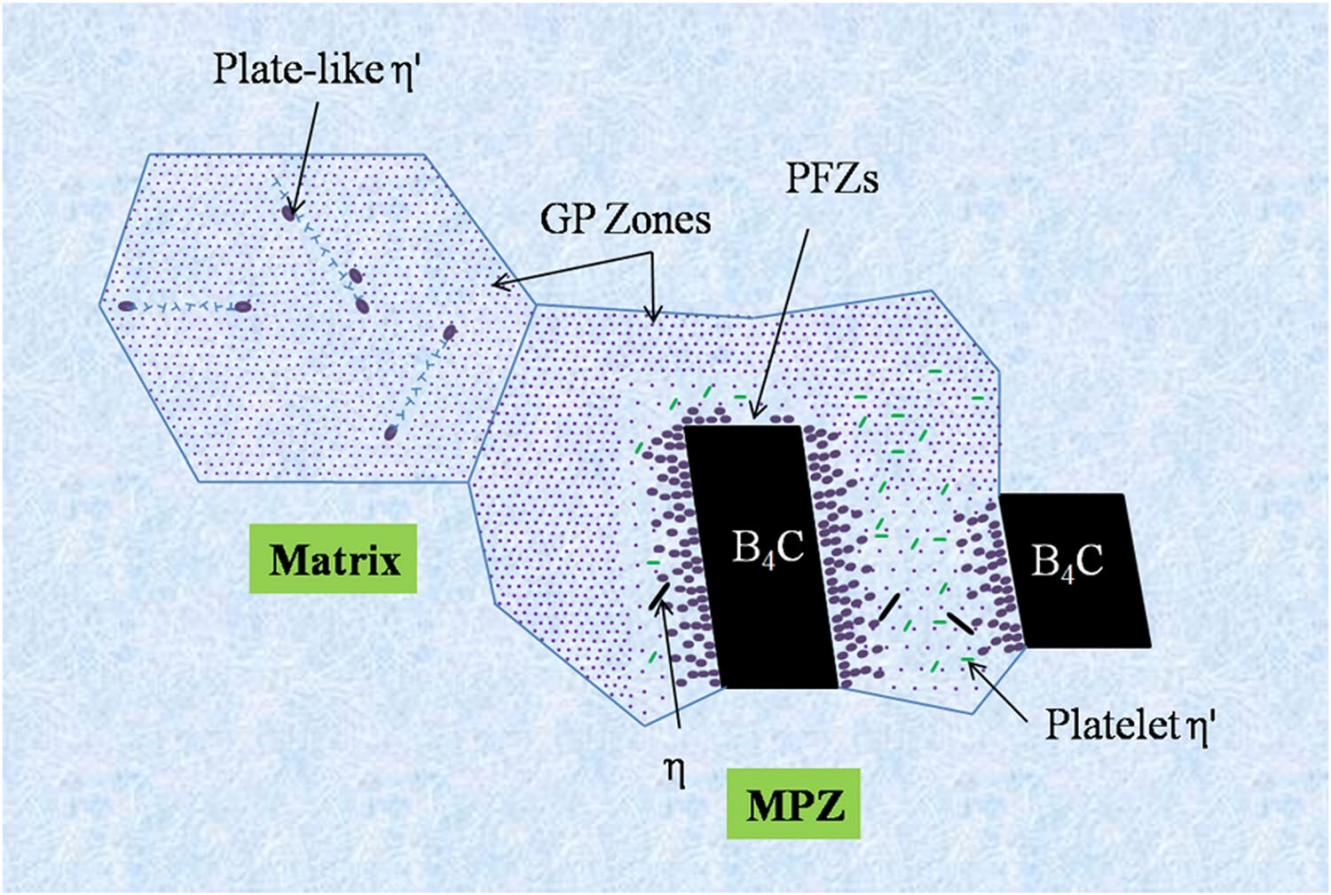
Schematic illustration indicating the distribution of various precipitates in matrix and MPZ.

In this section, an in-depth discussion on the formation mechanisms of the precipitates in different regions is provided, including schematic illustrations and proposed mechanisms. The discussion addresses nucleation of GP zones and η′ precipitates because these represent the predominant phases contributing to the strengthening in T6 tempered Al-Zn-Mg alloys via either Orowan dislocation bypassing or dislocation shearing mechanisms^16, 17, 43^. GP zones and η′ precipitates often form during the early stages of precipitation^14^. Proper control of early-stage precipitation, especially the size and number density of these precipitates, is therefore crucial to obtain optimal properties of the alloys^14, 44^. Our investigation aimed to elucidate the differences in precipitation behavior, in terms of the type, morphology, size, number density and dominant nucleation mechanism between the matrix and the MPZ. Comparisons of the characteristics of the precipitates are summarized in Table 1. Based on the aforementioned results, a schematic diagram is constructed (Fig. 8) to illustrate the distribution of the various precipitates in the two different zones in the composite.

**Table 1:** Summary of type, morphology, average size, number density and possible dominant nucleation mechanism of the precipitates in the Al 7075/B_4_C composites.

	type	Morphology	Average size (nm)	Number Density	Dominant nucleation and formation mechanism
MEZ	GP zone	Spherical	3.7	High	Homogeneous nucleation
	η′	Plate-like	58.7	Medium	Dislocation assisted nucleation
MPZ	GP zone	Spherical-like	4.7	Medium	Homogeneous nucleation
	η′	Plate-like	25.1	High	Dislocation assisted nucleation
		Platelet	11.6(length) 2.1(width)	Low	Heterogeneous nucleation
	η	Lath-like	110.4(length) 22.0(width)	Low	Heterogeneous nucleation

### Homogeneous nucleation and growth of GP zones

The homogeneous nucleation of the precipitates in Al-Zn-Mg alloy has been reported to be closely correlated to the concentration and distribution of vacancy sites^45^. Nevertheless, how dislocations influence GP zones remains the topic of some debates. Some earlier published results indicated that dislocations acted as the annihilation sites for vacancies^46, 47^. The nucleation of GP zones is influenced by solute-vacancy interactions^14^. During the initial stages of the aging treatment, quenched in vacancies interact with solute atoms such as Mg or Zn, then tend to form vacancy-solute clusters^34^ due to the attractive force existing between a vacancy and a solute atom. When the vacancies meet the solute atoms, the solute-vacancy pairs, including Mg-vacancy, Zn-vacancy and Zn_2_-vacancy, form and migrate in the matrix. The solute-vacancy clusters are subsequently trapped in the GP zones. The vacancies then virtually eliminated from the matrix after contributing to the process of GP zone formation. The rate of the nucleation of GP zone are closely correlated to the motion of Mg atom in the initial stages of the aging treatment because the activation energy of GP zone formation in Al-Zn-Mg alloy (0.62 eV) is close to the Mg activation energy (0.6 eV)^48^. Nevertheless, in the later stages, Zn atoms are considered to be more important than Mg atoms. This is related to the fact that the activation energy for growth of GP zone and their transformation into the η′ phase is about 0.32 eV^49^, which is close to that of the Zn-vacancy (0.42 eV)^50^ and Zn_2_ vacancy (0.21 eV)^51^. Ma *et al*. also reported that the Zn is the diffusion-rate-limiting solute atom during coarsening based on the calculation of bulk diffusivity^15^. Therefore, the nucleation and growth of GP zones requires a critical vacancy concentration during aging.

The lower number density of GP zones in MPZ could be ascribed to the annihilation of vacancies. Two possible reasons are proposed for this: (i) the Al/B_4_C interface, and (ii) dislocations, which can be detected from the bright field images in Fig. 7. Multiple studies have confirmed the formation of the dislocations caused by the CTE in the composites. For example, a High Voltage Electron Microscope (HVEM) equipped with a double tilt heating stage was used to characterize generation of dislocations at Al/SiC interface, the results indicated that the ceramic particles acted as dislocation sources during cooling from annealing temperatures^52^. Related research reported that dislocation densities in the Al-Zn-Mg(-Cu) alloys appeared to be lower when compared to those of the SiC particle reinforced composites^53^. Most of the previous researches also reported that grain boundaries, interfaces and dislocations act as sinks to absorb vacancies and caused annihilation^45, 47, 54, 55^. Papazian suggested that the composites with higher dislocation density exhibited more annihilation sites for vacancies, which in turn reduced the number of GP zones formed in the composite^19^. The tendency to lower the total Gibbs energy of the systems drives the vacancies to move to the sinks. Accordingly, the presence of dislocations and Al/B_4_C interfaces will facilitate the annihilation of vacancies, and therefore hinder the nucleation and growth of GP zones during aging. Moreover, GP zones serve as nuclei for the growth of η′ phase. The transformation from GP zones to platelet η′ also lead to the lower number density of GP zones in the MPZ. Once the nucleation of the GP zones occurs, the subsequent growth (coarsening) of the GP zones depends on the diffusion-rate-limiting species. Most of the previous research indicated that the dislocations act as fast paths for atomic diffusion^26^. The interaction between solutes and dislocations lead to a solute flux towards dislocations. This in turn resulted in a high concentration of solute atoms on/near the dislocations and subsequently facilitated the coarsening of the GP zones in the MPZ. Several dislocations were observed in the vicinity of GP zones (Fig. 3a). The Mg and Zn solute atoms diffused towards those dislocations can contribute to the growth and coarsening of GP zones. This suggestion is consistent with the prior published studies^34, 56^.

### Heterogeneous nucleation and growth of η′ precipitates

#### Platelet η′ precipitates

Interestingly, the platelet η′ precipitates were only observed in the MPZ. It is worth noting that when discussing the platelet η′ precipitates, the nucleation and growth of GP zones should also be taken into consideration. The precipitation sequence in the Al-Zn-Mg alloys commences with the formation of GP zones, which eventually transform into metastable η′ phases. The fine platelet η′ precipitate is the form of plate-like η′ precipitate in the early stage of development^34^. As increasing amount of Mg and Zn atoms diffuse to the platelet η′ during aging, some of the platelet η′ grows to the plate-like η′. Thus the platelet η′ is considered to be an intermediate phase^14, 40^. As discussed before, the concentration of vacancies in the matrix is much higher than that in the MPZ. The higher number density of GP zones (Figs 2d and 4d) could be attributable to the presence of the relatively higher concentration of vacancies in the matrix. The nucleation and growth of GP zones cause the depletion of solute atoms and thus hinder the transformation of the GP zones to η′ phases. Hu *et al*. also observed that the region containing higher number density of GP zones in CG7075-ET6 sample was devoid of platelet η′ precipitates as compared to that in UFG7075-ET6 sample^34^. The coherent interfaces of GP zones and the matrix have very low energies, however, small elastic coherency strains exist in the Al matrix^57^. As these coherency strains grow, the elastic energy associated with them is reduced by the formation of semi-coherent zones where dislocations form at the interface to take up the misfit strain. As shown in Fig. 5b, dislocations were observed in the inverse FFT images. In related work, Li *et al*. reported that defects exist in the structure of metastable η′ precipitates by means of high-resolution electron microscopy (HREM)^58^. These defects contribute to the formation of metastable η′ precipitates. Our present studies have confirmed that the preferable orientations for platelet η′ precipitates to nucleate are parallel to the (‐1‐11) and (‐11‐1) plane, which is consistent with results in refs^34, 35^. However, it is still unclear how the crystallography of the precipitates evolves during the transformation from coherent GP zone to semi-coherent η′ precipitates^14^.

#### Plate-like η′ precipitates

Our results reveal that the plate-like η′ precipitates show a relatively higher number density in the MPZ relative to that in the matrix, with a concomitant decrease in size. The plate-like η′ precipitates are noted to be associated with dislocation structure. According to related research, the main features of the heterogeneous precipitation on dislocations can be summarized as follow^2, 26, 34, 59, 60^: (i) nucleation sites: strain field associated with dislocations provide the driving force for the nucleation of precipitates, thus the dislocations could act as the preferred sites for heterogeneous nucleation, (ii) solute collector: dislocation motion attracts solute atom from the matrix because of the elastic interaction, the migration of solute atom causes the formation of solute-enriched regions on dislocation. (iii) pipe-diffusion path: dislocations are fast path for the diffusion of solute atom, which accelerate the coarsening of the precipitates nucleated on dislocations.

The majority of the plate-like η′ precipitates distributed on or near the dislocations, suggesting that the dislocations assisted the nucleation and growth of these precipitates. The precipitation process usually involves two stages: (i) nucleation and growth of precipitates during the early stages and (ii) coarsening of precipitates. During the nucleation and growth of the precipitates, the dislocations act as the preferred sites for heterogeneous nucleation^61^. During the early stage of development, the nucleation activation energy of platelet η′ precipitates on dislocation decreases compared to that required for homogeneous nucleation. The nucleation can be regarded as a standard heterogeneous nucleation law for sites with a linear density of *1*/*b* ^2^. The nucleation of the precipitates on the dislocation can be estimated as follows^2^:

1 $$\frac{d{N}_{d}}{dt}=\frac{4\pi ZD{C}_{0}{({R}_{0})}^{2}}{{a}^{4}b{(\mathrm{ln}({C}_{dis}/{C}_{eq}))}^{2}}\exp (-\frac{{\rm{\Delta }}{G}_{dis}}{{(\mathrm{ln}({C}_{dis}/{C}_{eq}))}^{2}})\exp (-\frac{{\tau }_{dis}}{t})$$

where ${R}_{0}=\frac{2\gamma {v}_{at}}{kT}$, *N*_*d*_ is the precipitate density, *Z* is Zeldovich’s factor (~0.05), *D* is the diffusivity of solute atom, *C*_0_ is the average solute concentration of the matrix, *C*_*eq*_ is the equilibrium solute concentration of the matrix, *C*_*dis*_ is the current solute concentration at the dislocations, *a* is the lattice parameter, *b* is Burgers vector (0.286 nm for FCC metals), *v*_*at*_ is the atomic volume (considered as constant for all species), *τ*_*dis*_ is the incubation period of nucleation at the dislocations.

The relationship of activation energy between homogeneous nucleation inside the matrix and heterogeneous nucleation along the dislocations could be defined as^62, 63^:

2 $$\frac{{\rm{\Delta }}{G}_{dis}}{{\rm{\Delta }}{G}_{hom}}=f(\alpha )=1-{\alpha }^{0.58}$$

where $\alpha =2\phi {\rm{\Delta }}{G}_{\alpha \beta }/\pi {\gamma }^{2}$ with $\phi =Gb/(\frac{4\pi }{1-v})$ for edge dislocations and $\phi =Gb/4\pi$ for screw dislocations. *G* is the elastic shear modulus (26.9 GPa for Al 7075), *γ* is the interfacial energy of the boundary, and *v* is the Poisson ratio (0.33 for Al 7075). Based on the qualitative analysis, the activation energy of the heterogeneous nucleation along the dislocations is lower than that of the homogeneous nucleation inside the matrix. In summary, the dislocation cores act as preferential nucleation sites for the plate-like η′ precipitates. It is feasible that the MPZ, containing higher density of dislocations, provides more heterogeneous nucleation sites during the early stage of the development. In addition to dislocations, Al/B_4_C interfaces in the MPZ also provide favorable nucleation sites for the η′ precipitates. As evidenced in Fig. 4, multiple plate-like η′ precipitates are observed in the vicinity of B_4_C particles.

After the nucleation of the platelet η′ precipitates, the subsequent growth and coarsening of the precipitates are closely correlated to the diffusion of the solute atoms. Details including the enhancement mechanism for growth and coarsening on the dislocation relative to the bulk diffusion could be found elsewhere^15^. The MPZs contain a higher density of dislocations (Fig. 7), and thus provides more heterogeneous nucleation sites. Thus, a higher amount of platelet η′ forms at the early stage. Once the size of the platelet η′ exceed the critical nucleus size, they continue to grow and coarsen to the plate-like η′ at the later stage. The depletion of solute atoms in the MPZ due to the higher number density of the platelet η′ at the early stage, hindered the further coarsening of the plate-like η′ precipitates.

#### Lath-like η precipitates

Several lath-like η precipitates were observed in the vicinity of B_4_C particles in the present study. Although prior studies suggested that GP zones and η′ precipitates are the predominant precipitates in T6 tempered 7xxx Al alloys^64^, the addition of the B_4_C reinforcement in the composite could accelerate the aging kinetics in the alloy matrix compared to that in the unreinforced counterparts^12, 65, 66^. The presence of B_4_C particles in the composites caused strain concentration and non-uniform distribution of dislocations at the Al/B_4_C interface and in the matrix region close to the interface as discussed in the previous section. Those dislocations could provide pipe diffusion paths for the solute atoms and accelerate the aging corresponding kinetics^9^.

## Conclusions

In the present study, the precipitation behavior in the matrix and the MPZ of age-hardening Al-Zn-Mg(-Cu) alloy matrix composites has been investigated by TEM and STEM. Based on the experimental observation and analysis, the following conclusions are made:

1. The precipitation phenomenon in the MPZ is different from that in the matrix. The GP zones and the plate-like η′ precipitates are the predominant phase in the matrix, however, in addition to these two phases, platelet η′ precipitates and lath-like η precipitates are also observed in the MPZ.
2. GP zones in the MPZ show a lower number density relative to that in the matrix, with a concomitant increase in the size. The lower number density of GP zones in MPZ could be hindered by the annihilation of vacancies and the transformation of GP zones to η′ phases. During coarsening, the GP zone with lower number density in MPZ could be provided by more solute atoms collected by the dislocations.
3. The nucleation and growth of GP zone in the matrix cause the depletion of solute atom and thus hinder the transformation of the GP zones to platelet η′ precipitates. Therefore, the semi-coherent platelet η′ precipitates can be only found in MPZ.
4. The activation energy of heterogeneous nucleation on the dislocation is decreased compared to the homogeneous nucleation. Thus, the dislocation cores act as preferential sites for the nucleation of the plate-like η′ precipitates. The dislocations combined with Al/B_4_C interface provided more heterogeneous nucleation sites for plate-like η′ precipitates in MPZ. It is the fact that the nucleation and growth of a high number density of η′ precipitates cause rapid depletion of the matrix solute. The growth and coarsening of the plate-like η′ precipitates in MPZ are hindered due to the relatively low supersaturation of solute atoms. Thus, the plate-like η′ precipitates with a relatively higher number density in the MPZ is much smaller than that in the matrix.

## Methods

### Materials preparation

Commercial Al 7075 alloy (gas-atomized; Al-5.2 wt.% Zn-2.28 wt.% Mg-1.53 wt.% Cu-0.21 wt.% Cr, prepared by Bai Nian Ying, Zhejiang, China) was chosen as the representative matrix alloy in the present study. B_4_C particles (Aladdin^TM^) were selected as the reinforcement phase. The median particles diameters of the two powders were 29.3 μm and 2.0 μm, respectively. A powder mixture with a target weight fraction of 92.5 wt. % Al 7075 and 7.5 wt. % B_4_C were blended in shaker-mixers for 24 h. After blending, the mixture was placed in a graphite mold without pre-pressing. PAS (ED-PAS III) was carried out in vacuum by heating to 530 °C at a rate of 100 °C/min, and then held for 3 min followed by cooling to room temperature in the furnace. The sintering pressure applied was 20 MPa. Additional details related to the PAS experiments can be found in the literature^31, 33^. Sequential heat treatments were performed at 466 °C for 2 h followed by a temperature increase up to 480 °C in 1 h, then quenched in water at room temperature and artificially aged at temperature of 120 °C for 24 hours^31^.

### Microstructural and elemental characterization

The composition of Al 7075 alloy was analyzed using an Inductively Coupled Plasma-Optical Emission Spectr (Prodigy 7). The median particles diameters were measured using a Malvern laser particle size analyzer (Mastersizer 2000). Samples for TEM studies were prepared by mechanically grinding the bulk material to a thickness of ~80 µm, then dimpling in the center to a thickness of approximately 15 µm. Further thinning to a thickness of electron transparency was carried out using an Ion Milling (Gatan PIPS 691) system at liquid nitrogen temperatures. The microstructure of the samples was characterized by transmission electron microscopy (JEOL2800, JEOL2500 and FEI CM20).

The number density of GP zones per unit area was estimated by counting the numbers of GP zones in at least ten STEM photographs of each zone using an image analysis tool, Image J^®^. The area for each photograph is approximately 160 nm × 160 nm.
